# A satellite cell‐dependent epigenetic fingerprint in skeletal muscle identity genes after lifelong physical activity

**DOI:** 10.1096/fj.202500177R

**Published:** 2025-03-06

**Authors:** Kevin A. Murach, Davis A. Englund, Toby L. Chambers, Cory M. Dungan, Hunter L. Porter, Jonathan D. Wren, Willard M. Freeman, Esther E. Dupont‐Versteegden, Yuan Wen

**Affiliations:** ^1^ Molecular Muscle Mass Regulation Laboratory, Department of Health, Human Performance, and Recreation, Exercise Science Research Center University of Arkansas Fayetteville Arkansas USA; ^2^ Department of Medicine University of Alabama at Birmingham Birmingham Alabama USA; ^3^ Department of Health, Human Performance, and Recreation Baylor University Waco Texas USA; ^4^ Genes & Human Disease Program Oklahoma Medical Research Foundation Oklahoma City Oklahoma USA; ^5^ Oklahoma City Veterans Affairs Medical Center Oklahoma City Oklahoma USA; ^6^ University of Kentucky Center for Muscle Biology Lexington Kentucky USA; ^7^ Department of Physical Therapy University of Kentucky Lexington Kentucky USA; ^8^ Department of Physiology University of Kentucky Lexington Kentucky USA; ^9^ Division of Biomedical Informatics, Department of Internal Medicine University of Kentucky Lexington Kentucky USA

**Keywords:** DNA methylation, methylome, stem cells, wheel running

## Abstract

Satellite cells comprise a small proportion of mononuclear cells in adult skeletal muscle. Despite their relative rarity, satellite cells have critical functions in muscle adaptation, particularly during prolonged exercise training. The mechanisms by which satellite cells mediate skeletal muscle responsiveness to physical activity throughout the lifespan are still being defined, but epigenetic regulation may play a role. To explore this possibility, we analyzed global DNA methylation patterns in muscle tissue from female mice that engaged in lifelong voluntary unweighted wheel running with or without satellite cells. Satellite cells were ablated in adulthood using the tamoxifen‐inducible Pax7‐DTA model. Compared to sedentary mice, wheel running for 13 months caused muscle DNA methylation differences in the promoter regions of numerous muscle fiber‐enriched genes—*Cacgn1*, *Dnm2*, *Mlip*, *Myl1*, *Myom2*, *Mstn*, *Sgca*, *Sgcg*, *Tnnc1*, *Tnni2*, *Tpm1*, and *Ttn*—only when satellite cells were present. These genes relate to muscle fiber identity, cytoarchitecture, and size as well as overall muscle function. Epigenetic alterations to such genes are consistent with previously observed histological and in vivo impairments to running adaptation after satellite cell depletion in these same mice. *Musk* promoter region methylation was affected only in the absence of satellite cells with lifelong running relative to sedentary; this dovetails with work showing that satellite cells influence skeletal muscle innervation. Defining the epigenetic effects of satellite cells on identity genes in muscle fibers after lifelong physical activity provides new directions for how these rare stem cells can promote muscle adaptation and function throughout the lifespan.

## Graphical abstract

Muscle stem cells (satellite cells) promote adult skeletal muscle adaptation. The presence of satellite cells affects the skeletal muscle methylome response to lifelong wheel running ‐ specifically influencing muscle fiber identity‐related genes. Impaired adaptation to lifelong wheel running without satellite cells may in part be explained by muscle epigenetic dysregulation.

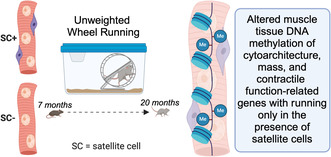

Skeletal muscle stem cells, or satellite cells, are essential for sustained hypertrophic responses to exercise training in adult skeletal muscle.^1^, ^2^ Genetic depletion of satellite cells inhibits muscle adaptation to voluntary wheel running in adult mice, even when accounting for reduced running volume in the absence of satellite cells.^1^, ^2^ Impaired long‐term adaptation to activity in adult muscle without satellite cells likely occurs via several mechanisms. First, abolishing myonuclear accretion may destabilize the “myonuclear domain” of growing muscle fibers, compromising overall myonuclear transcriptional capacity and subsequent muscle hypertrophy.^3^ Second, using single nucleus RNA‐sequencing, we found that the absence of satellite cells in adult mice leads to the emergence of transcriptionally “cryptic” resident myonuclei.^4^ These cryptic myonuclei lack a clear skeletal muscle fiber identity after weighted wheel running and emerge coincident with reduced adaptation.^4^ In context with our previous work showing distinct epigenetic contributions of resident versus satellite cell‐derived myonuclei to muscle adaptation in young adult skeletal muscle,^5^ we hypothesized that: (1) despite their relative rarity, satellite cells would generate a unique epigenetic signature during lifelong physical activity that is detectable at the tissue level, and (2) this signature may be enriched for genes linked to skeletal muscle identity.

The design of our study is depicted in Figure 1A. Satellite cells were ablated using our tamoxifen‐inducible Pax7‐DTA mouse model (see Methods and citations 1, 2, 4, and 5). Principal Component Analysis shows the global differences in DNA methylation between satellite cell‐replete and depleted muscles at ~20 months of age (Figure 1B). Satellite cell‐replete samples clustered more closely together than depleted samples, suggestive of a more consistent methylation profile mediated by the presence of satellite cells. In the same mice used in the current investigation, we previously reported how satellite cell depletion at five months of age followed by lifelong unweighted wheel running resulted in lower muscle mass, muscle fiber size, and in vivo muscle function by ~20 months of age.^2^ ~300 000 comparisons from the arrays impeded our ability to identify significance according to adjusted *p* values. We therefore utilized a stringent unadjusted *p* value cutoff (*p* < .0005) to identify CpGs of interest, in line with our previously published work.^6^ In the presence of satellite cells, gastrocnemius muscles of lifelong wheel running relative to sedentary mice were characterized by differences in promoter region methylation of skeletal muscle‐enriched genes. Overrepresentation analysis of differentially methylated CpGs in combined proximal and distal promoter regions returned “hypertrophic cardiomyopathy” (adj. *p* = .0007, KEGG) and “muscle contraction” (adj. *p* = .037, Reactome) as the top hits in satellite cell‐replete muscle after running. In proximal promoter regions (<1000 bp from a transcription start site, or TSS), CpGs in Calcium channel, voltage‐dependent, gamma subunit 1 (*Cacng1*, *p* = .0003, adj. *p* = .24), Dynamin 2 (*Dnm2*, *p* = .0003, adj. *p* = .24), Muscular LMNA interacting protein (*Mlip*, *p* = .0001, adj. *p* = .24), Myosin light chain 1 (*Myl1*, 3 distinct CpGs, all *p* < .0005 and adj. *p* = .24), Myostatin (*Mstn*, *p* = .0003, adj. *p* = .24), Sarcoglycan alpha (*Sgca*, *p* = .00049, adj. *p* = .24), Sarcoglycan gamma (*Sgcg*, *p* = .0002, adj. *p* = .24), Tropomyosin 1 (*Tpm1*, *p* = .0001, adj. *p* = .24), and Titin (*Ttn*, *p* = .0004, adj. *p* = .24) genes were hypermethylated in the muscle of lifelong running versus sedentary satellite cell‐replete mice (*p* < .0005) (Figure 1C, Table S1). Similarly, in distal promoter regions (1000–5000 bp upstream of TSS), *Dnm2* (*p* = .0003, adj. *p* = .24), Myomesin 2 (*Myom2*, *p* = .0003, adj. *p* = .24), *Sgca* (*p* = .00049, adj. *p* = .24), Troponin C1 (*Tnnc1*, *p* = .0003, adj. *p* = .24), and Troponin I (*Tnni2*, *p* = .0004, adj. *p* = .24) had hypermethylated CpGs after lifelong wheel running in mice with satellite cells (*p* < .0005) (Figure 1D, Table S2). These muscle identity‐related genes that are known to be enriched in muscle fibers were not different in their promoter regions with lifelong running compared to sedentary in the absence of satellite cells (Figure 1C,D, Tables S1 and S2).

**FIGURE 1:**
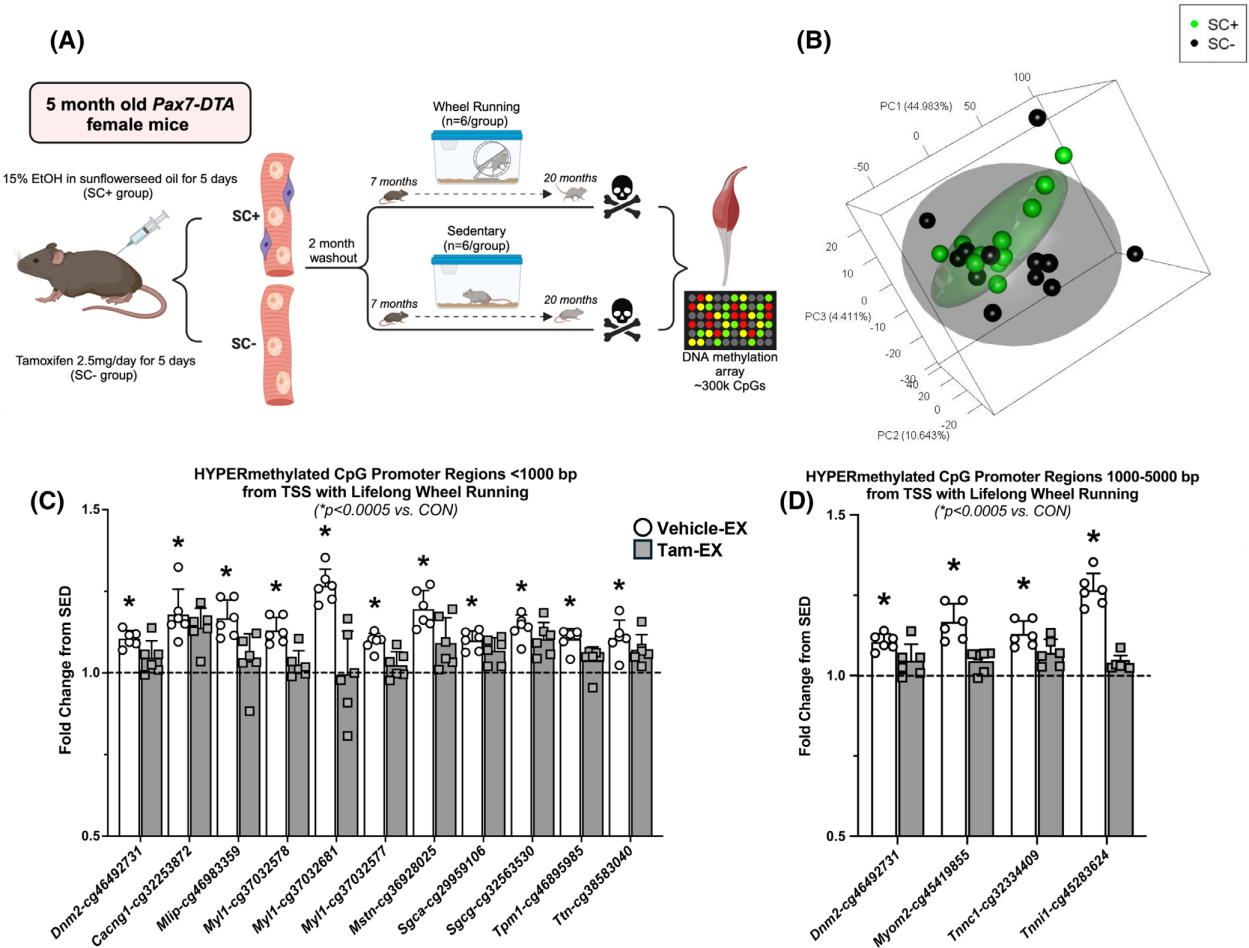
Epigenetic contributions from satellite cells to lifelong wheel running in gastrocnemius muscle of female mice. (A) Study design from Englund et al.^2^ (B) PCA plots of methylation data from satellite cell replete (SC+, *n* = 12) and deplete (SC−, *n* = 12) mice. The top 3000 most variable CpGs were used to generate the PCA plot. (C) DNA methylation profile of promoter regions (<1000 bp from TSS) after lifelong wheel running in the presence (SC+) versus absence (SC−) of satellite cells. (D) DNA methylation profile of promoter regions (1000–5000 bp from TSS) after lifelong wheel running in the presence versus absence of satellite cells. *p < .0005, fold difference for each individual sample was calculated as the difference between the average for the respective sedentary control group (line at 1.0 on the *y*‐axis in C&D).

Overrepresentation analysis of differentially methylated CpGs in proximal and distal promoter regions of satellite cell‐depleted muscle after lifelong running returned “cardiac conduction” (adj. *p* = .04, Reactome) as the top hit. The associated genes were related to ion transport (*Atb1b1*, *Fxyd1*, *Fxyd7*, *Kcnk2*, *Scn3a*) and not necessarily specific to muscle (Tables S1 and S2). The only skeletal muscle‐enriched gene with a promoter CpG that differed with running in the absence of satellite cells was muscle‐associated tyrosine receptor kinase (*Musk*). This gene is necessary for establishing skeletal muscle innervation patterns. *Musk* had a hypermethylated CpG <1000 bp from the TSS in running (0.55 ± 0.02, mean ± std. dev) versus sedentary muscle (0.48 ± 0.04) of satellite cell‐depleted mice (*p* = .0001, adj. *p* = .17, Table S1). Recent evidence suggests that myogenic progenitors influence nerve cells and that this is dependent on aging and exercise status.^7^ Satellite cell depletion in mice may also impair neuromuscular junction integrity throughout the lifespan.^8^ This methylation difference in the *Musk* gene in satellite cell‐depleted muscle with running could therefore be related to a lack of nerve‐satellite cell interactions. Worth mentioning is that CpGs in proximal and distal promoter regions of the type 1 collagen gene (*Col1a1*) were only altered in the absence of satellite cells (*p* = .0002, adj. *p* = .17, Tables S1 and S2). A lack of satellite cells affects fibrogenic cell behavior and *Col1a1* production,^9^, ^10^ so differential methylation in muscle tissue in the absence of satellite cells with running could be driven by fibrogenic cells. However, myonuclei produce appreciable *Col1a1* when muscle is loaded,^11^ so the source of this methylation difference with running in the absence of satellite cells deserves further consideration.

The effect of satellite cells on muscle fiber‐enriched gene methylation suggests that the epigenetic adaptations we report primarily reflect changes within myonuclei. Myosin light chains, troponins, tropomyosins, and titin are essential components of the skeletal muscle fiber cytoarchitecture and contractile machinery. We also speculate that some of the methylation differences observed in the presence of satellite cells could be fiber type‐specific since a few of the affected genes after running are characteristic of slow or fast‐contracting fibers (e.g. *Myl1*, *Tnnc1*, *Tnni2*, *Tpm1*). We cannot definitively say whether their promoter region hypermethylation stems from resident or satellite cell‐acquired myonuclei in the presence of satellite cells. A recent study showed that loading‐induced muscle hypertrophy without satellite cell fusion altered the expression of “muscle development and differentiation” genes in resident myonuclei.^12^ Furthermore, 72 h of mechanical overload—a time point prior to satellite cell fusion—in satellite cell replete plantaris muscles of young mice resulted in lower *Myl1* (adj. *p* = .05), *Mstn* (adj. *p* = .07), *Sgca* (adj. *p* = .0003), *Sgcg* (adj. *p* = .02), and *Tpm1* (adj. *p* = .001) specifically in myonuclei relative to sham muscle.^11^ Resident myonuclei are likely responsible for the methylation signature we observe.

Hypermethylated promoter CpGs in muscle‐enriched genes in the presence of satellite cells could relate to reduced gene expression—either at baseline or following a bout of exercise—as this is generally the result of promoter region hypermethylation. For example, promoter region hypermethylation of a CpG in Myostatin—a negative regulator of muscle mass—aligns with lower *Mstn* mRNA in the plantaris muscle at the same time point after running only in the presence of satellite cells (−0.1% difference in satellite cell depleted versus −6.0% in satellite cell replete), concomitant with muscle growth in these same mice.^2^ Promoter region hypermethylation of other muscle identity genes after exercise only in the presence of satellite cells may seem counterintuitive; however, we recently reported that both late‐life exercise training and partial epigenetic reprogramming of skeletal muscle by Yamanaka Factors in satellite cell replete mice were characterized by lower mRNA levels of some muscle identity genes, including *Tnni2*, which was likely transient.^6^ Thus, satellite cells may in part mediate the “rejuvenating” molecular signature and qualities of exercise in skeletal muscle. On balance, muscle DNA methylation is complex and dynamic after exercise in skeletal muscle and could oscillate between hyper‐ and hypomethylation dependent on the recovery timepoint. Our analysis at 48 h post‐exercise^2^ could therefore differ from methylation patterns that may occur at earlier recovery timepoints (e.g. <24 h), which tend to align best with gene expression.^13^ For instance, the muscle methylome profile at 30 min post‐exercise in humans is most aligned with gene expression at 3 h of recovery, but not later recovery time points out to 24 h.^9^ Given this complexity, further research is needed to define the specific transcriptional outcomes of methylation status dependent on satellite cells with physical activity throughout the lifespan.

Our experiments indicate that satellite cells establish a distinct promoter region methylation fingerprint in skeletal muscle following lifelong wheel running that likely contributes to enhanced muscle adaptation and contractile function compared to satellite cell‐depleted mice.^1^, ^2^ In order to find statistical significance according to adjusted *p* value for individual CpGs, future investigations should utilize a larger sample size and perhaps more targeted analyses involving fewer comparisons. Nevertheless, our findings complement previous work on the unique transcriptional contributions of satellite cells to muscle loading^1^, ^4^, ^5^, ^12^ and lay the groundwork for future studies examining cell type‐specific epigenetic regulation of adult muscle adaptation.

## 1 METHODS

This study was approved by the IACUC at the University of Kentucky, and all procedures were performed in accordance with the NIH *Guide for the Care and Use of Laboratory Animals*. At five months of age, female Pax7‐DTA mice were injected intraperitoneally with vehicle (15% ethanol in sunflower seed oil) or tamoxifen (2 mg/day, suspended in ethanol and sunflower seed oil) for five days, followed by a two‐month washout period, then assigned to sedentary or wheel running groups—satellite cell replete (vehicle) or depleted (tamoxifen). Comprehensive muscle and activity phenotypes for these mice are reported in Englund et al.^2^

Methylation analysis was performed with Illumina Mouse Methylation assays at the Oklahoma Nathan Shock Center. Methylation array data were processed from images to methylation matrices by methylprep (https://github.com/FoxoTech/methylprep) and preprocessed by removing sex chromosome probes and probes with known SNPs, retaining only CpG (cg) and CH (ch) probe types. Differential methylation analysis was conducted using the limma package in R. A linear model was fitted to the beta values using a design matrix that accounted for both exercise conditions (Sedentary/Exercise) and treatment (Vehicle/Tamoxifen). Empirical Bayes moderation was applied to compute moderated t‐statistics and *p*‐values across all measured CpGs. CpGs were mapped to all genomic features across ~300,000 sites using an annotated manifest file containing chromosomal locations and associated genes; promoter regions were focused on in this project. Overrepresentation analysis was performed using ConcensusPathDB in the mouse module using default settings and Reactome and KEGG databases, and *q* values (adj. *p*) are reported (http://cpdb.molgen.mpg.de/).

## AUTHOR CONTRIBUTIONS

Kevin A. Murach, Yuan Wen, and Esther E. Dupont‐Versteegden conceived the study analysis approaches. Kevin A. Murach, Hunter L. Porter, and Yuan Wen analyzed the data. Kevin A. Murach and Yuan Wen wrote the manuscript. Toby L. Chambers generated the figures. Kevin A. Murach, Davis A. Englund, and Cory M. Dungan managed and/or performed experiments. Kevin A. Murach, Yuan Wen, Davis A. Englund, and Esther E. Dupont‐Versteegden provided resources, oversight, and/or intellectual contributions. All authors provided feedback and final approval of the manuscript.

## FUNDING INFORMATION

This work was supported by the National Institutes of Health (NIH) R00 AG063994 KAM and R00 AR081367 and R01 AG069909 to YW. This research was conducted while KAM was a Glenn Foundation for Medical Research and AFAR Grant for Junior Faculty awardee. This work was supported by a Nathan Shock Center Pilot Grant from the Oklahoma Medical Research Foundation to KAM, YW, and EED‐V. WMF was supported by NIH IK6BX006033. WMF, JDW, and HLP were supported by NIH P30AG050911.

## DISCLOSURES

YW is the founder of MyoAnalytics LLC.

## Supporting information


**Supplemental Table 1.** Proximal promoter region CpGs (<1000 bp from TSS) in SC+ and SC‐ running mice relative to sedentary controls, *p* < .0005. NA = Not Annotated. Data are presented as a proportion with 0 being completely hypomethylated and 1 being completely hypermethylated.
**Supplemental Table 2**. Distal promoter region CpGs (1000–5000 bp from TSS) in SC+ and SC‐ running mice relative to sedentary controls, *p* < .0005. NA = Not Annotated.

## Data Availability

Array data are deposited in GEO GSE290238.
